# Retraction Note: Cyanidin-3-glucoside activates Nrf2-antioxidant response element and protects against glutamate-induced oxidative and endoplasmic reticulum stress in HT22 hippocampal neuronal cells

**DOI:** 10.1186/s12906-024-04672-2

**Published:** 2024-10-07

**Authors:** Monruedee Sukprasansap, Pithi Chanvorachote, Tewin Tencomnao

**Affiliations:** 1 Food Toxicology Unit, Institute of Nutrition, Mahidol University, Salaya campus, 25/25 Phuttamonthon 4 Road, Salaya, Nakhon Pathom, 73170 Thailand; 2 Department of Pharmacology and Physiology, Faculty of Pharmaceutical Sciences, Chulalongkorn University, Bangkok, 10330 Thailand; 3 Cell-based Drug and Health Products Development Research Unit, Faculty of Pharmaceutical Sciences, Chulalongkorn University, Bangkok, 10330 Thailand; 4 Age-Related Inflammation and Degeneration Research Unit, Department of Clinical Chemistry, Faculty of Allied Health Sciences, Chulalongkorn University, Bangkok, 10330 Thailand

**Retraction Note: BMC Complementary Medicine and Therapies (2020) 20:46**

10.1186/s12906-020-2819-7

The Editor has retracted this article after concerns were raised about some of the data reported. In Fig. 4a there is evidence of potential splice marks in the Calpain blot, and there also appear to be several duplicated lanes within the ß-actin blot. The authors were unable to provide raw data for this figure upon request by the Publisher. The Editor therefore no longer has confidence in this article’s findings or conclusions.

The authors did not respond to correspondence from the Publisher about this retraction.

